# Dr. Benjamin H. Lemon

**Published:** 1906-01

**Authors:** 


Dr. Benjamin H. Lemon, of Thorold, Ont., died suddenly at his home, November 28, 1905, aged about 70 years. He was demonstrator of anatomy at the University of Buffalo, 1858-9, and was Coroner of the counties of Lincoln and Welland at the time of his death. He graduated at Victoria University in 1857.

